# The most common bone tumors of the upper extremity in childhood and adolescence and their treatment: a review of the current literature

**DOI:** 10.1530/EOR-2025-0149

**Published:** 2026-04-07

**Authors:** Dong Yu, Alexandre Kaempfen, Maximilian Burger, Kira Barlow, Andreas H Krieg

**Affiliations:** ^1^ Department of Orthopedics, University Children’s Hospital (UKBB), Basel, Switzerland; ^2^ University of Basel, Basel, Switzerland; ^3^ Bone and Soft Tissue Tumor Center of The University of Basel (KWUB), Basel, Switzerland; ^4^ Plastic, Reconstructive, Aesthetic and Hand Surgery, University Hospital Basel, Basel, Switzerland

**Keywords:** upper extremity, growing pain, benign bone tumor, malignant bone tumor, reconstruction

## Abstract

- In upper extremity bone tumors, osteochondromas and solitary bone cysts represent the predominant benign entities, with osteosarcoma accounting for the majority of malignant presentations. The proximal humeral metaphysis emerges as the most prevalent anatomical site across both tumor entities.
- Upper, one-sided extremity pain in children and adolescents should be followed up and diagnosed, since ‘growing pain’ in the upper extremities is not a common finding.
- Osteochondromas should be surgically addressed early if they impose a risk of development of a deformity, such as those located on the forearm and the distal tibia, where they can cause growth disorders and thus functional impairments.
- Reconstructions for pediatric malignant bone tumors of the upper arm or forearm should allow the spatial placement of the hand.
- Given the longevity of sarcoma survivors, the longevity of the reconstruction is an important planning consideration. Biological reconstructions combining autologous/vascularized bone with tendon repair and transfers appear to be the most appropriate and preferable to prosthesis whenever possible.
- Multidisciplinary collaboration involving plastic surgeons with hand reconstruction expertise constitutes a critical component in orthopedic oncology treatment planning.

## Introduction

This article discusses the common types of upper limb bone tumors in children and adolescents under the age of 18, presenting the clinical manifestations, diagnostic methods, and various treatment options for benign and malignant bone tumors based on the latest data from the Swiss National Reference Center for Bone Tumors. The bone tumors discussed in this article originate from the upper limb skeleton, including the humerus, radius and ulna of the forearm, as well as the carpal, metacarpal, and phalangeal bones of the hand.

## Incidence

The types of tumors and their prevalence listed in this article are based on data from the Swiss National Reference Center for Bone Tumors as of May 2025 and are interpreted in the context of established international literature. While registry data provide invaluable insights into regional trends, it is acknowledged that the overall incidence and distribution of pediatric bone tumors can vary geographically.

**Figure 1:**
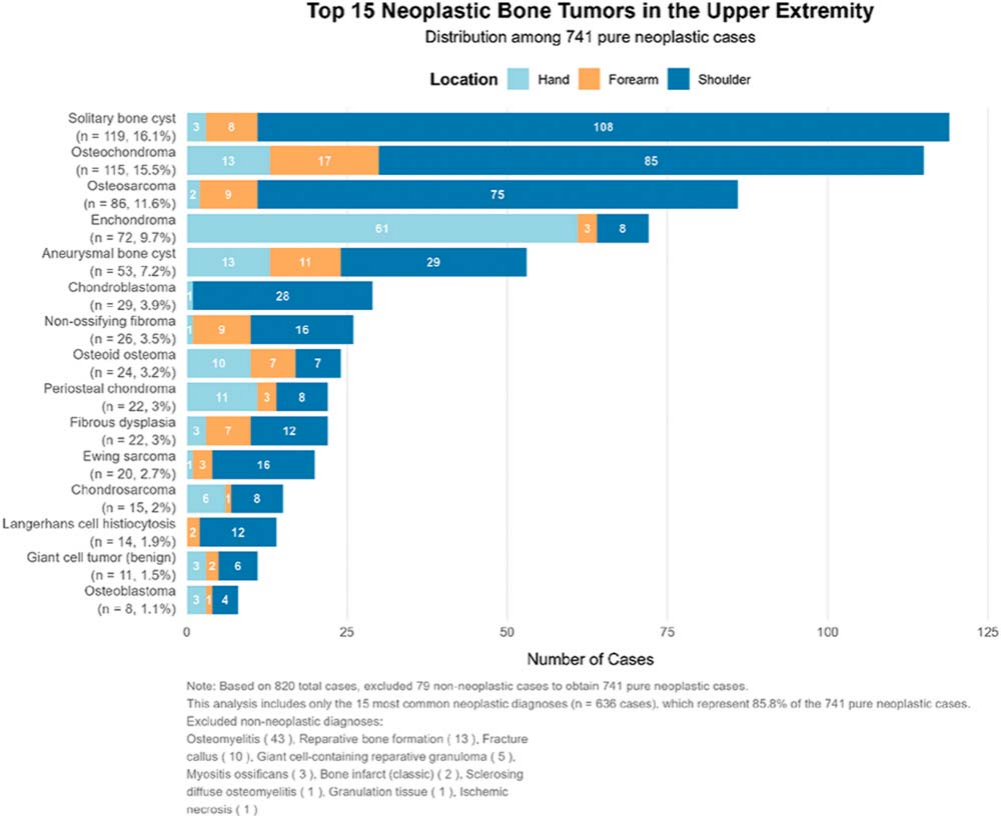
Frequency and localization of malignant and benign bone tumors on the upper extremities: humerus, forearm (radius and ulna), and hand (carpal bones, metacarpals, and phalanges) – current data from the National Bone Tumor Reference Center (KTRZ) of the University of Basel.

Globally, osteochondromas and simple bone cysts are consistently reported as the most common benign bone tumors (1, 2), a finding that is strongly reflected in our national cohort where they represent the predominant benign entities in the upper limb (Fig. 1). The most common benign bone tumors in the upper limb are osteochondromas and solitary bone cysts, with the latter most frequently occurring in the proximal humerus. However, it must be considered that solitary bone cysts are probably underreported, as they are not commonly biopsied and often treated conservatively (3, 4). Compared to the lower limb, non-ossifying fibromas are relatively rare in the upper limb, although the number of not detected cases in biomechanically less stressed arms and forearms must be significantly higher.

**Figure 2:**
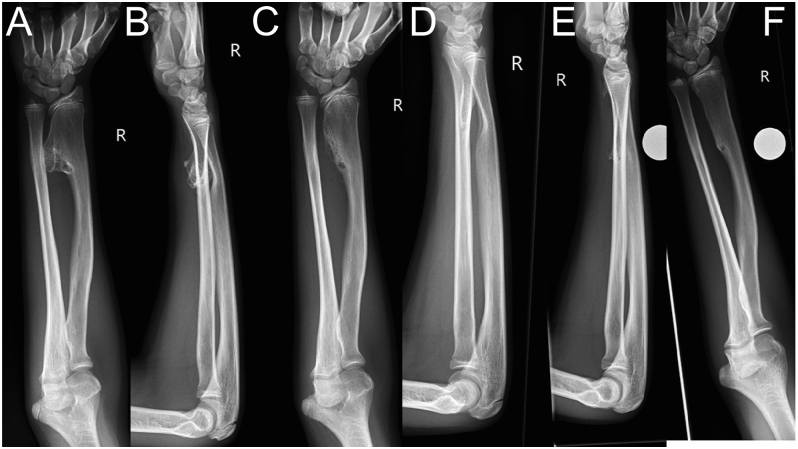
(A and B) Twelve-year-old girl with increasing deformity and right wrist pain caused by a large radial osteochondroma displacing the ulna; (C and D) 2 months later after simple resection, the axis and joint ratios have been normalized; (E and F) at 18-month follow-up, functioning is normal.

In the humerus, chondroblastomas and aneurysmal bone cysts are more common, but the most common bone tumors in the forearm are enchondromas, particularly in the phalanges of the hand. Overall, the forearm has a relatively lower incidence of tumors compared with the proximal humerus and the hand, with osteochondromas representing the most frequent benign lesion. Notably, while an osteochondroma in the proximal humerus does not usually cause growth disturbance, lesions in the forearm are almost always associated with growth disturbances (Fig. 2A).

In malignant bone tumors, osteosarcomas remain the most common subtype in the pediatric population worldwide (5), and our registry confirms their predominance in the upper extremity, primarily located in the proximal humerus. Ewing sarcoma is typically the second most common malignant bone tumor in children and adolescents across all skeletal sites (6), which is consistent with our data, where it follows osteosarcoma and is mainly found in the humeral shaft. Other malignant bone tumors are extremely rare and only appear in the upper limb in isolated cases (Fig. 1) in our cohort. Malignant bone tumors in the hand are rarer than in other parts of the upper limb. Giant cell tumors, as intermediate tumors, require multimodal treatment if they present with rare bony manifestations in the wrist and metacarpal regions.

## Clinical presentation and diagnosis

Unlike the lower limb, non-specific unilateral pain in the upper limb of children is less common and should be taken seriously. Only wrist pain may occasionally occur in this age group, often associated with minor trauma. ‘Growing pains’ are not a recognized entity in the upper limb (7). A detailed clinical history and examination are paramount. Night pain or localized pain in the upper limb is only seen in rare cases of benign tumors, such as osteoid osteoma (8) and osteoblastoma (9). Osteosarcomas present with constant intermittent pain (10). Therefore, for upper limb pain in children and adolescents, routine biplanar X-rays should be taken, and there should always be a valid reason for not conducting such imaging.

The diagnostic workflow for a child with persistent upper limb pain should always begin with biplanar X-rays. If these are negative or inconclusive but clinical suspicion remains high, further imaging is mandatory.

For suspected benign tumors, such as osteoid osteoma/osteoblastoma, CT scan is the gold standard for characterizing the nidus and planning ablation, despite radiation concerns in children (11). In the case of osteochondroma, MRI is indicated to assess the cartilage cap thickness (a value >2 cm in skeletally mature patients raises concern for secondary chondrosarcoma) and for preoperative planning to evaluate relations to neurovascular structures (12). For aggressive benign/intermediate tumors (e.g., ABC and GCT), MRI is essential to define the intramedullary and soft tissue extent and for differential diagnosis (11).

For suspected malignant tumors, international guidelines from the Children’s Oncology Group (COG) and the European Society for Pediatric Oncology (SIOP) recommend a standardized approach (13, 14): plain radiographs: initial study showing aggressive patterns (e.g. permeative destruction, Codman’s triangle, and sunburst periosteal reaction); whole-limb MRI: the cornerstone of local staging (it must include the entire involved bone to exclude skip metastases and should be performed with contrast to assess neurovascular involvement and intramedullary extent); and CT chest and PET-CT/bone scan: for systemic staging. Biopsy remains the gold standard and must be performed by an experienced orthopedic oncologist, adhering to a planned future surgical approach to minimize the risk of contamination.

## Bone cysts

**Figure 3:**
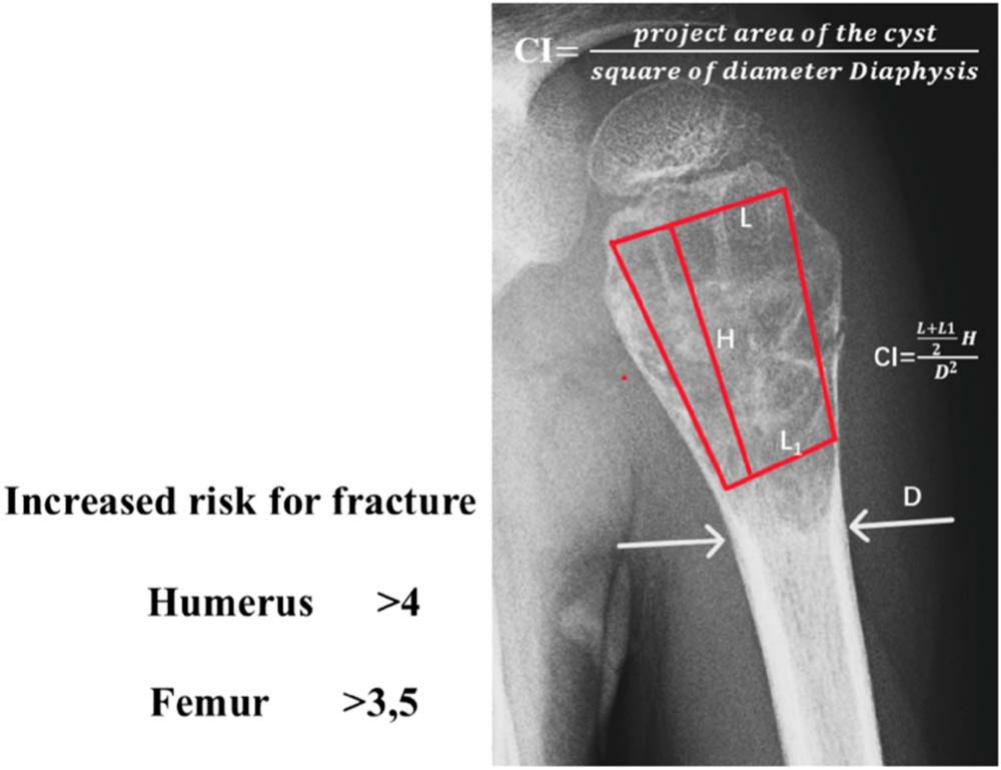
Kaelin & MacEwen found that the larger the cyst, the more cortex was destroyed and the bone was weakened. To quantify the strength of the remaining cortex, which depends on the size of the cyst and the size of the affected bone, they developed the cyst index. This indicates the relationship between the radiographic area of the cyst and the size of the affected bone, measured as the square of the diameter of the diaphysis (19).

Most upper limb bone tumors are diagnosed incidentally or following a pathological fracture, particularly solitary bone cysts, which often occur in the proximal humerus (15). In older textbooks, solitary bone cysts are still considered self-limiting tumor-like lesions that typically do not require treatment, as they may heal spontaneously after a fracture or ossify after growth cessation (16). All lesions previously classified as ‘tumors of uncertain nature’ have been reclassified as true tumors according to the new WHO classification since 2020 (17). For example, recurrent translocations of the NFATC2 gene have been identified in simple bone cysts, although their significance remains unclear (18). However, no malignant manifestations were observed in the clinic, and we consider it to be benign, although it needs to be evaluated. Plain X-rays can assess the fracture risk of solitary bone cysts using the cyst index (19) (Fig. 3).

## Osteochondromas

Osteochondromas in the upper arm typically become clinically detectable only when they reach critical dimensions, particularly within the proximal posteromedial humeral region. This delayed presentation may relate to mechanical irritation of the brachial plexus secondary to shoulder rotation or adduction maneuvers. Clinical assessments frequently emphasize the pathognomonic protuberance palpated during physical examination, potentially overlooking associated neuropathic manifestations. Therefore, we recommend thorough neurological examinations in patients with upper humeral osteochondromas.

It is important to note that while osteochondromas of the proximal humerus are common and often asymptomatic, they can lead to mechanical issues, such as tendinitis, limitations of shoulder motion, tendon ruptures, and neurovascular compression (20). The decision for prophylactic excision in this location is less clear-cut than in the forearm but should be considered if serial imaging demonstrates progressive deformity or if the patient experiences significant pain or functional limitation.

In the forearm, osteochondromas cause significant growth disturbances in 30–60% of cases (Fig. 2A) (21, 22). Particularly in the forearm, rotational dysfunction caused by osteochondromas or the tumor’s impact on hand function is the main issue. Fortunately, most lesions are solitary and often involve the periosteum of the radius. Approximately 15% of osteochondromas are associated with multiple hereditary exostoses. Bilateral involvement exacerbates functional loss. About 1% of osteochondromas may undergo malignant transformation into chondrosarcoma. This usually involves trunk tumors rather than those in the arm (23).

Osteochondromas were previously classified according to the Masada classification (18), but this classification only covers possible deformities in the forearm and has not been validated. The newer Jo classification also addresses important bilateral osteochondroma lesions in the distal forearm, making it a more suitable classification system (24). However, this classification is also considered to have only moderate to fair reliability by experts (25). In the hand, osteochondromas may cause tendon snapping, finger crossover, or nail deformities due to bony protrusions and growth plate irritation.

## Enchondromas

Enchondromas are the most common bone tumor of the hand (26), leading to phalangeal thickening and occasionally causing pathological fractures in adolescents. However, without trauma, they typically do not cause pain in the humerus and forearm, but more often in the smaller bones of the fingers. In cases associated with Maffucci syndrome or Ollier’s disease, the incidence is higher, particularly in the hand (27). More frequent pathological fractures in these cases help to early diagnose the disease.

When biopsy is considered for an equivocal central cartilaginous lesion, it should be interpreted with caution. In cartilaginous tumors, biopsy may be unreliable for definitive grading and assessment of malignant potential, in part due to histological overlap and sampling limitations. Therefore, biopsy findings must be integrated with clinical and imaging features by a specialized multidisciplinary team to guide management (28).

## Malignant bone tumors

Malignant bone tumors in the upper limbs of children, such as osteosarcoma or Ewing sarcoma, are rare but demand high clinical suspicion.

**Figure 4:**
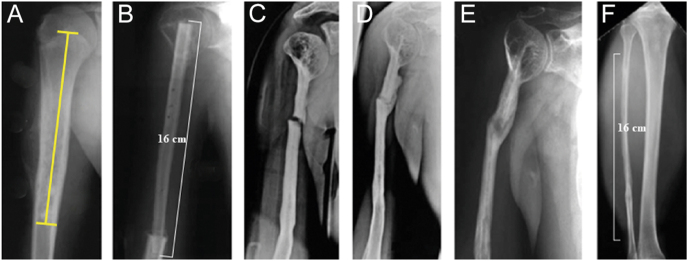
Sixteen-year-old boy with chondrosarcoma of the right humerus; (A) preoperative anteroposterior radiograph; and (B) postoperative radiograph after further resection (R0) and implantation of a non-vascularized fibula graft. The fibula was wedged only in the proximal and distal parts of the humerus; (C) traumatic transverse fracture of the graft 6 years after reconstruction. Conservative treatment consisted of an upper arm brace; (D) good callus formation after 3 months; (E) 26.5 years after the operation with integration and remodeling of the transplant; (F) complete regeneration of the fibula.

**Figure 5:**
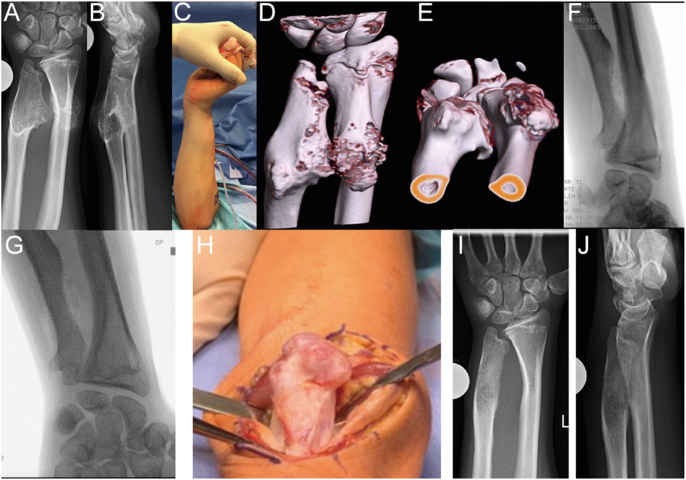
(A, B, C, D, E) Eighteen-year-old adolescent boy with left-sided osteochondroma; (F and G) resection of the large osteochondroma on the left wrist in the supine position (dominant); (H) intraoperatively; (I and J) 6 months postoperatively with restored turning movement and congruence in the distal radioulnar joint.

Persistent nocturnal or resting pain is one of their characteristics and is frequently accompanied by localized swelling and elevated skin temperature (29). X-ray may demonstrate aggressive bone destruction (lytic lesions and sunburst periosteal reactions) or soft tissue masses (Fig. 4A). MRI is essential for assessing tumor extent and neurovascular invasion (Fig. 5A). Biopsy remains the gold standard for diagnosis and must be performed by an orthopedic oncology specialist to minimize the risk of iatrogenic seeding. Differential diagnoses include osteomyelitis and Langerhans cell histiocytosis.

Early referral to a specialized orthopedic oncology center is critical. Treatment planning requires integration of histopathological findings with a multidisciplinary evaluation.

## Treatment

### Benign bone tumors

#### Solitary bone cysts

**Figure 6:**
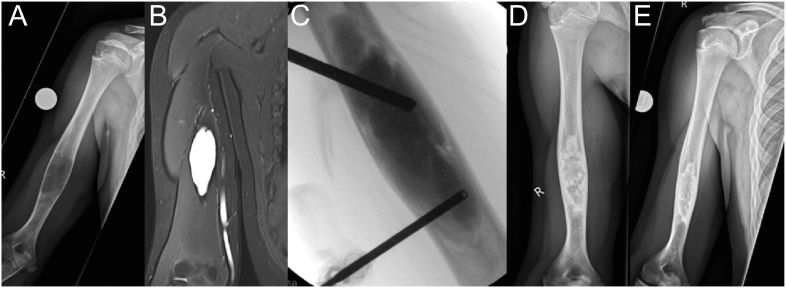
(A and B) Twelve-year-old boy with a solitary bone cyst, symptomatic presumably due to stress fracture and increased fracture risk; (C) percutaneous cyst aspiration and filling with bioresorbable bone cement; (D) no recurrence at 8-month follow-up; (E) no recurrence at 34-month follow-up.

Based on our own observations, one-third resolve after fracture healing. Persistent ones may be absorbed after growth cessation (80% of cases) or remain as asymptomatic residuals. The issue arises when they continue to enlarge during childhood, increasing the risk of recurrent fractures; this risk can be assessed using the cyst index (Fig. 3). According to the literature, spontaneous healing after a fracture occurs in only about 10–15% of cases (30). In such cases, prophylactic stabilization is recommended, such as orthotic immobilization or intramedullary fixation (31), complex curettage with autologous material filling (32), or percutaneous aspiration with absorbable bone substitute filling (Fig. 6) (33).

The treatment of solitary bone cysts (SBCs) remains debated, as highlighted by multicenter studies such as those from the EPOS group (34). The primary aims of any intervention should be i) definitive cyst control to prevent recurrence, ii) minimization of complications (e.g. growth arrest and iatrogenic fracture), and iii) enabling a rapid return to normal activity for the child. Since both complex surgery and minimally invasive percutaneous procedures yield similarly good outcomes for this benign and potentially self-limiting tumor, we prefer the latter approach.

#### Osteochondromas

Most osteochondromas in the upper limb are treated similarly to those in other skeletal regions. Surgical removal is only necessary if pain or functional impairment occurs (21), as they are not a malignant condition, and there is a possibility of spontaneous regression (35). In the forearm, rotational dysfunction due to osteochondromas is common. Whether prophylactic removal of osteochondromas should be performed to prevent this remains controversial. In particular, forearm shortening can be corrected through lengthening procedures (21).

In our series of 17 forearms (15 patients) with a follow-up period of 20 years, four limbs were managed with observation alone, while 13 underwent surgical treatment. Approximately one-third of the patients underwent tumor resection alone, while the rest also underwent bone realignment or lengthening. At 20-year follow-up, 85% of the excision-only cohort demonstrated enhanced wrist/elbow range of motion versus 70% improvement rates observed in patients undergoing concurrent limb-lengthening procedures (elbow stiffness was observed in two patients). In addition, forearm lengthening surgery is associated with the use of fixators, which may lead to severe complications. Four out of six patients required unplanned reoperations. The non-surgical group had more significant functional and cosmetic deficits compared to the surgical group. The non-surgical group had more significant functional and cosmetic deficits compared to the surgical group. This aligns with the growing international consensus that favors earlier intervention for forearm osteochondromas to prevent complex deformities (36, 37).

Based on these experiences, we recommend early resection of forearm osteochondromas before functional impairment occurs (Fig. 2). Lengthening surgery is only considered in cases of radial head dislocation, as this procedure permanently impairs rotational function, and long-term radial head resection may lead to ulnar impaction at the wrist (Fig. 5).

Similarly, the approach to finger lesions should follow this principle. Tumors causing growth abnormalities and affecting finger function (even adjacent fingers) should be resected early.

In the distal phalanx, timely tumor resection can restore normal phalangeal growth and reliably alleviates pain.

#### Enchondromas

Enchondromas can occur throughout the upper limb. In the fingers and metacarpals, they are often diagnosed through X-rays following minor trauma due to excessive pain. Treatment is usually unnecessary, as conservative healing processes, such as splinting for fractures, can achieve sufficient filling and healing (24).

**Figure 7:**
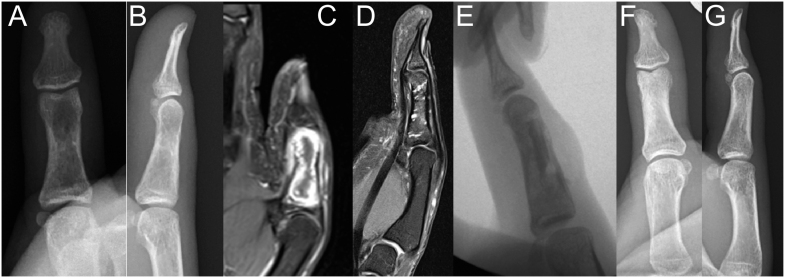
Filling of multiple enchondromas with cement in the lateral and dorsal part; (A, B, C, D) preoperatively; (E) intraoperatively; (F and G) 1 year postoperatively.

For recurrent painful trauma or larger lesions, curettage with autologous cancellous bone or substitute material filling is advisable (Fig. 7). Mechanical instability rarely requires fixation, whether internal or external. For progressively enlarging lesions, such as in Ollier’s disease, diagnosis should be confirmed through a complete curettage to clarify the condition. In the context of multiple enchondromatosis, the lifetime risk of secondary chondrosarcoma transformation is significantly elevated (up to 40% in Maffucci syndrome) (38). In such cases, any new onset of pain, rapid enlargement, or concerning features on MRI (e.g. cortical destruction and soft tissue mass) should prompt a biopsy. A peripheral (centrifugal) biopsy technique, sampling the most aggressive-looking area (including the periphery and any soft tissue component), is crucial for obtaining diagnostic tissue and differentiating enchondroma from chondrosarcoma.

### Other tumor entities of the upper extremity

Locally aggressive, but benign tumors (for example, aneurysmal bone cysts, periosteal chondromas, and giant cell tumors) can typically be managed with curettage or en bloc resection and reconstruction. Biological bridging or filling with vascularized or non-vascularized autografts is usually sufficient. For aneurysmal bone cysts, neoadjuvant treatment options (such as cryotherapy, sclerotherapy, or radiofrequency ablation) (32) can also be considered. In cases of articular involvement, osteochondral allografts demonstrate durable functionality in upper extremity applications where biomechanical demands are comparatively reduced, typically maintaining efficacy until degenerative articular changes necessitate definitive arthrodesis procedures (39).

### Malignant bone tumors

The management of upper limb sarcomas requires a multidisciplinary team. The surgical challenge lies in achieving wide oncological margins while providing a functional and durable reconstruction. Table 1 summarizes the common surgical reconstruction options, their indications, and reported outcomes.

**Table 1:** Overview of surgical reconstruction options for malignant upper extremity bone tumors in children. Data synthesized from the referenced literature and institutional experience.

Tumor location	Surgical procedure	Key indications	Reported functional outcomes (MSTS/ROM)	Advantages	Disadvantages and complication rates
Proximal humerus (deltoid sacrificed)	Glenohumeral arthrodesis (e.g. with vascularized fibula-allograft composite)	Unsalvageable deltoid/axillary nerve	Stable, painless shoulder. 30–90° thoracoscapular elevation. Good hand/elbow function. MSTS: ∼70–80% (41)	High durability, low long-term failure rate	Non-union (10–20%), infection, scapular fracture. Loss of shoulder motion (42, 43)
Proximal humerus (deltoid intact)	Mobile reconstruction (e.g. APC, osteoarticular allograft, vascularized fibula epiphysis transfer)	Preserved deltoid/axillary nerve and glenoid	Active shoulder abduction/elevation possible. MSTS: ∼75–85% (47)	Preservation of joint motion. Growth potential with vascularized physeal transfer (50, 51)	Graft resorption/fracture, infection, instability/dislocation. High revision rates (up to 50% at 10 years for some techniques) (47, 49)
Diaphysis (humerus, forearm)	Intercalary biological reconstruction (vascularized/non-vascularized fibula, allograft)	Adequate residual epiphyseal bone for fixation	Excellent long-term union and remodeling potential. Near-normal function (50, 51)	Durable, biological integration	Stress fracture (non-vasc: up to 40%), non-union (51). Prolonged protection needed
Distal forearm/hand	Biological reconstruction (e.g. free vascularized bone graft, arthrodesis)	Preservation of basic hand function is a goal	Focus on stable, painless wrist or joint for grasp (52)	Maintains sensitivity, avoids prosthetic issues	Technically demanding. Risk of non-union, graft failure
	Amputation + prosthesis	Very extensive tumors where reconstruction is not feasible	Good basic function with modern prosthetics (55)	Definitive oncological control. No reconstruction complications	Rejection, sensory deficit (55), lifelong dependency on device. High cost of maintenance

APC, allograft-prosthetic composite; MSTS, musculoskeletal tumor society score.

When managing upper limb sarcomas, the surgical team faces the challenge of ensuring adequate resection margins while providing a reliable and durable reconstruction. Given the spatial constraints of pediatric anatomy, it is often not feasible to resect several centimeters of tissue around the tumor. Therefore, understanding which tissue layers serve as reliable tumor barriers and how to plan a reasonable and safe resection is crucial. Another important aspect of shoulder and proximal humerus sarcoma surgery is preserving or restoring hand function. Ultimately, optimizing shoulder and elbow function is another primary goal after reconstruction.

In our database, osteosarcoma is the most common. Most pediatric cases require intra- or extra-articular shoulder resection. However, en bloc resection of the scapula and lateral clavicle or extended resection according to the Tikhoff–Linberg classification (40) is usually unnecessary. Although many methods exist for reconstructing proximal humeral defects, most can be categorized into two types: glenohumeral arthrodesis or mobile joint reconstruction. The most important discrimination factor whether a joint reconstruction is feasible depends on the possibility to preserve a functional deltoid muscle, including an intact axillary nerve. When a functional deltoid cannot be preserved, scapulohumeral arthrodesis demonstrates superior functional outcomes compared to humeral prosthetic suspension techniques involving the glenoid fossa, clavicular anchoring, or costal anchoring (41). During arthrodesis, at least 30° of thoracohumeral elevation can be achieved through periscapular muscles, including the upper trapezius. Some children can achieve nearly 90° of flexion and abduction through adaptive spinal lateral bending.

**Figure 8:**
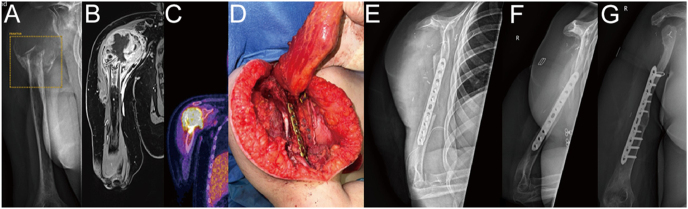
(A, B, C) Twelve-year-old girl with a high-grade osteosarcoma of the right proximal humerus (X-ray, MRI, and PET-CT); (D) intraoperatively; (E) 1 week postoperatively; (F and G) 1 year postoperatively.

Allograft transplantation is the traditional material for arthrodesis. Compared with the use of an isolated vascularized fibula, complications such as resorption, fractures, and infections can be reduced or possibly avoided by a composite graft (a double-barrel vascularized fibula graft within an allograft) (42, 43). Alternative options include a pedicled scapular pillar graft with allograft bone (44) or the clavicula pro humero technique (45). The latter requires medial separation and downward rotation of the clavicle to fuse with the humerus, suspending the upper extremity at the acromioclavicular joint (Fig. 8). In skeletally mature individuals, the length of the defect that can be restored is limited to approximately 12 cm, and non-union may be an issue. However, if the deltoid is intact, good functional elevation of 30°–90° can be achieved. The latter option is particularly useful when the deltoid can be preserved but the scapular neck and glenoid cannot (44).

Glenohumeral joint fixation primarily depends on deltoid function, but functional outcomes rely on restoring active rotation (glenohumeral/scapulothoracic). A key goal is achieving about 90° rotation from hand-to-belly to neutral, enabling most daily activities when combined with ≥30° abduction. Reattaching pectoralis major provides internal rotation, while transferring teres major/latissimus dorsi to the humeral head or plate can restore external rotation (46). If deltoid function is preserved, mobile reconstruction may be beneficial. Traditional options include osteoarticular allografts (42, 47) and processed autografts (irradiated/pasteurized) (47), but both carry high revision risks due to resorption, fracture, or infection.

It is reported that the outcome of arthroplasty is different and sometimes unsatisfactory and may be accompanied by other problems, such as dislocation (46). Allograft transplantation (or recycled bone) (48), allograft-prosthetic composite (APC) (47), arthroplasties, and osteoarticular allograft transplantation yield similar functional outcomes. The reported complication rates for reconstructions, such as APC and osteoarticular allografts, are significant. Revision rates can be as high as 26%, with infection being 6% and mechanical failure being 30% (49). However, APC has a lower tendency for fracture and revision.

In the humerus, forearm shaft, or when preserving the proximal and distal epiphyses, biological reconstruction with vascularized or non-vascularized autografts shows good long-term outcomes in children and adolescents (50, 51). In pediatric oncology, ‘good long-term results’ encompass multiple domains: i) high rates of overall survival and local disease control, ii) durability of the reconstruction without the need for multiple major revision surgeries, iii) preservation of acceptable function of the hand and elbow, and iv) good patient-reported outcomes regarding quality of life and body image. However, assessing these outcomes is challenging. As pediatric patients transition into adulthood, they often move to adult care systems, leading to loss to long-term follow-up. This makes it difficult to capture truly lifelong data on complications such as late degenerative changes, mechanical failure of biological reconstructions, or secondary malignancies. The remodeling potential in this age group is excellent, even for non-vascularized fibulas (Fig. 4).

Malignant tumors of the hand and distal forearm are rarer in our database. Particularly in the hand, reconstruction should consider the principles of basic hand function and acceptable appearance. Whenever possible, grasping function should be reconstructed for young patients. The so-called basic hand consists of a stable, controllable, and sensitive thumb, a broad grasping span, and a stable (if possible) sensitive pillar that the thumb can move against. Additional elements, such as mobility of the pillar or a second finger to achieve a three-point grip, are desirable but already represent functional improvements. Paco del Pinal defined these reconstruction goals in more detail in a 2006 review article (52). Techniques for this purpose include toe transplantation in children, free functional muscle transplantation, tendon transfer, and nerve reconstruction. For reconstructing joints in the hand, microsurgical techniques, such as toe joint transplantation, can be employed to restore motion (53). Smaller defects of bone can also be addressed with free vascularized bone grafts (such as the femoral condyle) or non-vascularized reconstructions (54).

Bionic prostheses are continually improving and may be a viable option for adult patients in some cases; however, they are less effective for children due to their weight, lack of sensitivity, and difficulty to control (55).

Skin and soft tissue defects in the hand and distal forearm should be reconstructed primarily whenever possible to prevent granulation tissue formation, which may encase many functional sliding structures. If primary reconstruction is not possible due to tumor diagnosis, negative pressure dressings can be used for a short period (less than 7 days) (56). Nerve reconstruction can also be performed with allografts. However, the current research landscape is still heavily influenced by manufacturers, and autograft reconstruction remains preferred (57).

For all young patients, avoiding multiple revisions and achieving long-term durability over decades is necessary to reduce dependence on arthroplasties. Many surgeons have made pioneering efforts in developing biological methods, which are promising alternatives (51). Combining biological bone reconstruction with tendon transfer and nerve grafting or transfer requires significant time and effort but can yield profound long-term benefits.

## Discussion

This overview, based on clinical data from the Swiss National Bone Tumor Reference Center, systematically analyzes the clinical characteristics, diagnostic strategies, and treatment options for upper extremity bone tumors in children and adolescents, providing critical insights for the clinical management of this unique population. Our findings highlight the distinct biological behavior and functional reconstruction challenges of upper extremity bone tumors and propose a personalized treatment framework centered on long-term outcomes.

### Biological and functional specificity of upper extremity bone tumors

This overview demonstrates that benign tumors in the upper limb are more prone to early nerve compression or functional impairment due to anatomical constraints. Malignant tumors demand advanced reconstruction plans to preserve limb function. These differences stem from the upper limb’s lower biomechanical load but higher functional precision, particularly in children, where preserving growth potential and restoring long-term joint mobility are necessary, but fine motor skills are prioritized.

Our data confirm the proximal humerus as a ‘hotspot’ for malignant tumors, requiring a balance between wide resection and shoulder function preservation. Some pediatric patients undergoing functional reconstruction following oncological resection achieve pseudo-flexion approximating 90° through kinematic compensation mechanisms involving adaptive lateral spinal flexion maneuvers. This functional reconstruction establishes a robust foundation for rehabilitation of daily living.

### Optimization and controversies in treatment strategies

#### Timing of proactive intervention for benign tumors

Our study challenges the universal applicability of the traditional ‘watch-and-wait’ approach. For forearm osteochondromas, early resection reduced the need for complex corrective surgeries, with the excision-only group showing superior 20-year functional improvement (85 vs 70% in combined lengthening procedures). These findings advocate revising current guidelines to include ‘functional warning signs’ as surgical indications.

For solitary bone cysts, we prioritize minimally invasive treatments: percutaneous injection of bioresorbable substitutes avoid open surgery. The Kaelin index (17) serves as a validated risk stratification tool, with an index value > 4.0 in upper extremity lesions indicating critical cortical weakening and warranting prophylactic surgical intervention to mitigate pathological fracture risks.

### Advantages of biological reconstruction in malignancies

In pediatric upper extremity malignancies, biological reconstruction (vascularized/non-vascularized autografts) demonstrated children’s remarkable bone remodeling capacity – even non-vascularized fibular grafts achieved. We further propose the ‘composite biological reconstruction’ concept: combining vascularized fibular grafts with allograft plates (double-barrel technique) while preserving joint reconstruction potential (toe transplantation (53, 58)).

Compared to APCs, biological reconstruction greatly avoids the possibility of refurbishment, although it demands higher technical expertise and multidisciplinary support, particularly from microsurgeons.

### Challenges in long-term outcome assessment

A critical, yet often underdiscussed, aspect of pediatric orthopedic oncology is the difficulty of long-term follow-up. The transition from pediatric to adult healthcare systems frequently results in patient attrition, making it challenging to obtain complete data on the lifelong performance of reconstructions, the rate of very late complications, or the true incidence of secondary malignancies. Prospective, multinational registries are essential to overcome this challenge and refine our understanding of the outcomes we report.

### Critical considerations in clinical decision-making

#### Hierarchical goals of functional reconstruction

This study establishes a prioritized model for upper extremity malignancy reconstruction: i) primary goal: preserve grasp and fine motor skills (hand function), ii) secondary goal: restore elbow flexion/extension and forearm rotation, and iii) tertiary goal: achieve shoulder stability or limited mobility. For example, in proximal humeral tumors with intact axillary nerve function, mobile reconstruction (e.g., vascularized fibular grafts) is preferred. Otherwise, arthrodesis with scapulothoracic compensation remains reliable (Fig. 8).

### Necessity of multidisciplinary collaboration

Our cohort’s success relied on integrated efforts among orthopedic oncologists, microsurgeons, and rehabilitation teams. Complex hand reconstructions, such as free toe transfers requiring simultaneous tendon rebalancing and sensory nerve coaptation, exemplify the need for seamless teamwork.

## Conclusion

The management of upper extremity bone tumors in children and adolescents requires balancing radical tumor control and functional preservation. This study confirms that personalized biological reconstruction strategies significantly improve long-term outcomes, with multidisciplinary collaboration as the cornerstone of success. Through technological innovation and multicenter collaboration, we aim to refine treatment standards, ultimately achieving the synergistic goals of ‘oncological cure’ and ‘functional restoration’.

## ICMJE Statement of Interest

The authors declare that there is no conflict of interest that could be perceived as prejudicing the impartiality of the research reported.

## Funding Statement

This research did not receive any specific grant from any funding agency in the public, commercial, or not-for-profit sector.
